# Machine learning and time-series approaches for forecasting bacterial blight of pomegranate

**DOI:** 10.3389/fpls.2026.1787474

**Published:** 2026-07-16

**Authors:** Manoj Choudhary, Niranjan Singh, Meenakshi Malik, Meenakshi Arya, P. N. Meena, S. M. Haldhar, Mukesh Khokhar, Yukti Chandervanshi, Umesh Saini, Niharika Shoeran

**Affiliations:** ICAR-National Research Institute for Integrated Pest Management, New Delhi, India

**Keywords:** ARIMA, bacterial blight, machine learning, pomegranate, SARIMA, time-series modelling

## Abstract

Bacterial blight, caused by *Xanthomonas axonopodis* pv. *punicae*, poses a significant threat to pomegranate (*Punica granatum* L.) cultivation, resulting in considerable yield losses. The disease exhibits pronounced seasonal variability, which is largely influenced by environmental and climatic factors. Understanding the temporal behaviour of disease severity and its relationship with meteorological factors is essential for effective disease surveillance, forecasting, and management. This study analysed long-term surveillance data on bacterial blight severity from pomegranate-growing regions in Maharashtra, India (2013–2024), along with meteorological parameters. This study employed statistical methods, time-series models (ARIMA, SARIMA, and VAR), and Machine learning (ML) based regression models using seasonal assessments of disease severity based on Standard Meteorological Weeks (SMWs). The results indicated weak associations between disease severity and weather variables, with temperature showing a weak positive correlation (r = 0.18), while relative humidity exhibited a moderate inverse relationship (r = −0.33). Time-series analysis revealed a clear temporal dependence in disease progression, with the non-seasonal ARIMA (2,1,1) model providing the best fit (R² = 0.691; RMSE = 0.085), whereas seasonal components were not retained in the final model. The multivariate VAR model further enhanced biological interpretability by integrating weather variables, achieving a comparable accuracy (R² = 0.732; RMSE = 0.239). Among ML–based regression models, LightGBM achieved the best prediction (R² = 0.776; RMSE = 0.566). The explainable ML analysis consistently identified temperature as the dominant driver of bacterial blight severity. Overall, the integrated analytical framework provides a robust understanding of bacterial blight dynamics by combining surveillance data, temporal modelling, and predictive analytics. These findings provide a basis for data-driven forecasting systems, enabling timely interventions and improved disease management under varying climatic conditions.

## 1 Introduction

Pomegranate (*Punica granatum* L.), popularly known as the “Fruit of Paradise,” is a nutritionally, medicinally, and economically important fruit crop belonging to the family *Lythraceae* (Manjunatha et al., 2024). Global pomegranate production exceeds 8 million tonnes annually, largely concentrated in India, China, Iran, and Turkey, which together contribute nearly 80% of the global production (Liu et al., 2025). In India, pomegranate cultivation has expanded markedly owing to increasing domestic demand and export potential. During 2021–22, the crop covered approximately 2.7 lakhs hectares, with a total production of 30.86 lakhs metric tonnes and an average productivity of 11.4 t ha^−1^ (Madhumathi et al., 2023). Maharashtra is the major pomegranate-producing state in India, accounting for over 80% of the total cultivated area (Kadbhane and Manekar, 2025). Pomegranate cultivation in Maharashtra during 2020–21 covered approximately 1.62 lakh ha, with a total production of 17.48 lakh metric tonnes, reflecting the state’s dominant contribution to the national pomegranate output (NHB, 2020-2021).

Pomegranate cultivation is severely constrained by biotic stresses, particularly diseases caused by fungal and bacterial pathogens, including *Alternaria alternata* (heart rot), *Aspergillus niger* (fruit and heart rot), *Botrytis cinerea* (grey mould), *Penicillium* spp. (blue mould), *Colletotrichum gloeosporioides* (anthracnose), *Ceratocystis* spp. (wilt), and *Xanthomonas axonopodis* pv. *punicae* (bacterial blight) (Munhuweyi et al., 2016). Among these, bacterial blight is the most destructive disease, affecting all aerial parts of the pomegranate plant and producing water-soaked lesions on leaves and fruits that progressively darken and become necrotic, often resulting in leaf drop and fruit cracking (Chathalingath and Gunasekar, 2023), thereby adversely affecting pomegranate yield and fruit quality.

Abiotic factors, such as temperature, rainfall, humidity, and moisture stress, influence pomegranate growth and productivity while simultaneously modulating the severity of bacterial blight. Bacterial blight in pomegranates persists across a broad temperature range (9–43 °C) under varying humidity conditions (30 to >80%); however, disease development intensifies during the rainy season due to the availability of free moisture (Sharma et al., 2015), which provides congenial conditions for bacterial multiplication. Although the pathogen can tolerate low humidity and extreme temperatures, severe disease outbreaks are consistently associated with high relative humidity (>80%) and moderate temperatures (25–35 °C) (Munhuweyi et al., 2016). Monsoon conditions from July to September, characterized by sustained humidity and optimal temperatures (25–30 °C), promote rapid pathogen multiplication and spread (Chathalingath and Gunasekar, 2023), leading to epidemic levels of disease. In contrast, during the pre-monsoon period (April–July), the disease generally remains mild to moderate because of high temperatures (Ashish and Arora, 2016).

To overcome the climate-driven challenges of bacterial blight, seasonal and temporal assessments of disease dynamics concerning meteorological drivers have been adopted. Integrating disease surveillance data with weather variables enables the identification of critical periods of disease risk and supports the development of forecasting and early warning systems (Wimberly et al., 2017). Statistical approaches, including correlation and regression analyses, are widely used to evaluate the combined influence of weather parameters on disease development and severity and to predict disease progression (Rehman and Atiq, 2024). Building on these static associations, time-series modelling offers an effective framework for characterizing the temporal dependency and seasonal reoccurrence in disease progression under fluctuating weather conditions (Loona et al., 2025). Autoregressive and seasonal models, such as Autoregressive Integrated Moving Average (ARIMA) and Seasonal Autoregressive Integrated Moving Average (SARIMA), have been widely adopted to describe disease severity based on historical trends and cyclical behaviour (Szostek et al., 2024). These models enable the identification of short-term persistence and recurring seasonal peaks in disease incidence (Peng et al., 2025). Patil et al. (2025) applied ARIMA and SARIMA models to analyse and forecast fruit rot disease severity caused by *Phytophthora meadii* in arecanut across three agroclimatic regions of Karnataka during 2018–2019, where ARIMA captured short-term disease fluctuations while SARIMA predicts severity (59.8%) more effectively, represented seasonal trends and provided longer-term disease forecasts. Similarly, Meena et al. (2024) employed an ARIMA-based modelling framework to predict rice blast severity caused by *Magnaporthe oryzae* using long-term weekly meteorological data collected from 2017–2023. The ARIMA model achieved high predictive accuracy (R² = 0.92) and demonstrated its utility for weather-driven disease forecasting and targeted disease management. Chittaragi et al. (2026) utilised SARIMA models to capture seasonal progression and forecast anthracnose severity caused by *Colletotrichum lagenarium* in bottle gourd during the monsoon seasons of 2023–2024, highlighting the suitability of seasonal time-series models for weather-linked disease prediction and early warning applications. In addition, multivariate approaches, such as Vector Autoregression (VAR), allow the simultaneous evaluation of disease progression and meteorological variables to examine their time-dependent interactions (Hossain et al., 2024). A long-term study based on sugarcane data from 2008–2023 identified effective cumulative temperature and rainfall as key meteorological drivers of sugar content accumulation after confirming stationarity using the Augmented Dickey–Fuller (ADF) test. These variables were incorporated into a VAR framework to analyse their dynamic and bi-directional temporal relationships with sugar content across regions and growth stages (Zheng et al., 2024).

Recent advances in Machine learning (ML) have enabled more accurate disease prediction under complex climatic variability by modelling nonlinear and high-dimensional disease and weather relationships (Chowdhury and Rahman, 2025). Models such as Random Forest (RF), Extreme Gradient Boosting (XGBoost), and Light Gradient Boosting Machine (LightGBM) are widely adopted in plant disease surveillance owing to their capacity to capture complex interactions and improve predictive robustness across diverse agroclimatic conditions (Mermer et al., 2025; Nyawose et al., 2025). For instance, Akhter and Sofi (2024) employed multiple ML models, including RF, XGBoost, and LightGBM, to predict bacterial blight severity in rice using environmental variables such as temperature, humidity, rainfall, and soil moisture, where XGBoost achieved the highest prediction accuracy (92.5%). In wheat, ML-based regression modelling has been applied to assess weather-driven disease severity, where RF models demonstrated strong predictive capability for yellow rust and powdery mildew, achieving R² values of 0.97–0.98 during calibration and 0.90–0.93 during validation (Bijlwan et al., 2025). Similarly, Dubey et al. (2025) applied RF, Cubist, XGBoost, and GBM models to predict yellow mosaic disease severity in yardlong beans, with the RF model emerging as the most reliable predictor under field conditions based on standardized ranking performance (sRPI = 1.00). Collectively, these studies demonstrate that ML-based ensemble and boosting algorithms are particularly effective for disease severity prediction and support their applicability in climate-driven disease forecasting frameworks.

Despite considerable advances in predictive modelling, studies integrating statistical analysis, time-series modelling, and ML within a unified framework remain limited. The present study addresses this gap by employing a multi-paradigm modelling approach comprising statistical, time-series models (ARIMA, SARIMA, and VAR), and ML-based methods, using long-term surveillance data with a comprehensive set of meteorological variables. The novelty of this study lies in the cohesive integration of diverse modelling paradigms within a single analytical framework applied to long-term time-series data, facilitating robust temporal analysis and improving the reliability and interpretability of forecasts. This framework further strengthens early warning capabilities and supports climate-informed and sustainable management of bacterial blight in pomegranate cultivation. The key contributions of this study are as follows:

- To provide a comprehensive analysis of bacterial blight severity dynamics in pomegranates across major growing districts of Maharashtra, India, using long-term surveillance data (2013-2024).
- To evaluate the temporal and seasonal patterns of bacterial blight progression and their association with key meteorological drivers under diverse agro-climatic conditions.
- To present and evaluate statistical time-series models alongside multiple ML models for long-term disease forecasting, demonstrating the applicability of an integrated modelling framework for plant disease studies.
- To conduct a data-driven disease forecasting to identify critical risk windows and associated meteorological determinants, facilitating timely risk assessment and informed management decisions for bacterial blight in pomegranate.

## 2 Research methodology

**Figure 1:**
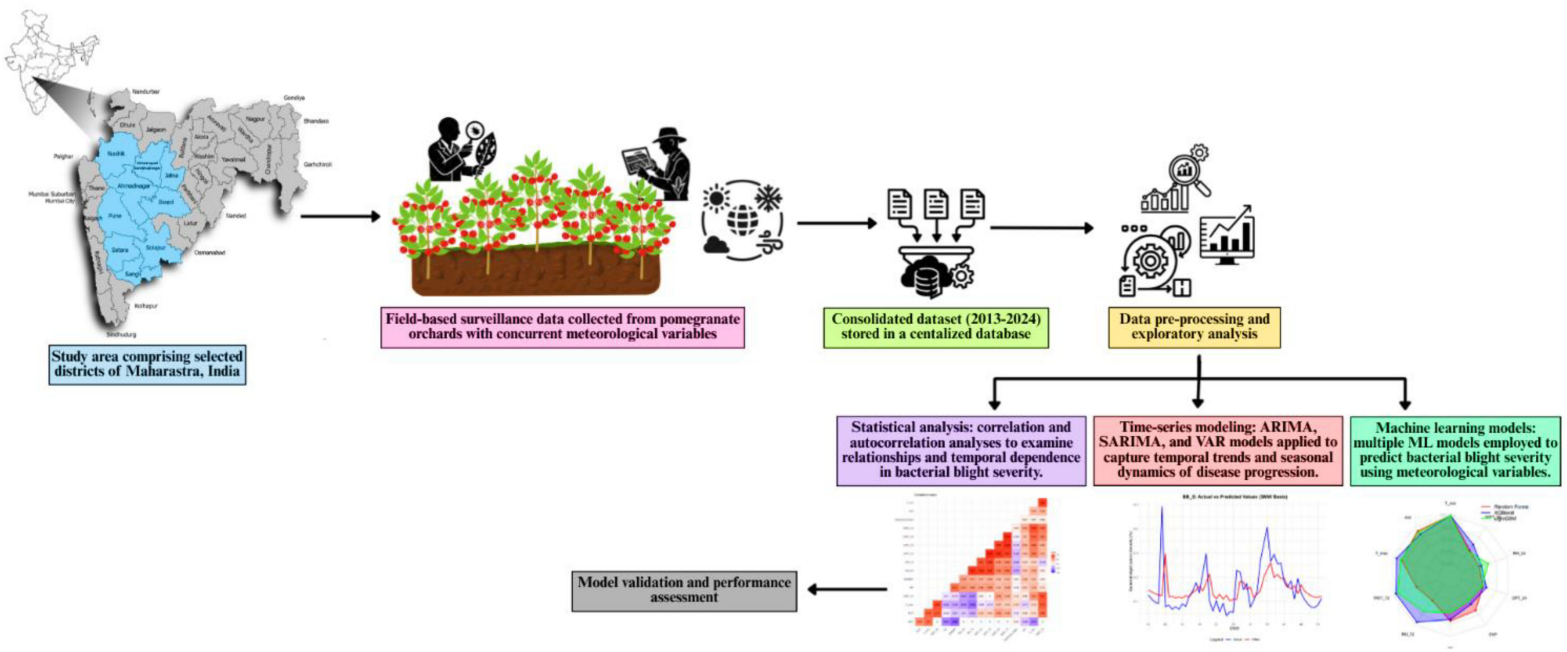
Schematic representation of the research workflow.

The proposed research methodology follows a structured analytical framework that begins with data collection, followed by data pre-processing and statistical analysis. Subsequently, time-series modelling, seasonal analysis, and ML models were applied to derive meaningful insights from the dataset. The overall workflow of this study is shown in **Figure 1**:

### 2.1 Study area & data acquisition

The study was conducted to assess bacterial blight disease severity in major pomegranate regions across nine districts of Maharashtra, India, including Ahmednagar, Beed, Chhatrapati Sambhajinagar, Jalana, Nashik, Pune, Sangli, Satara, and Solapur. These districts cover diverse agro-climatic conditions, enabling a comprehensive evaluation of disease trends. The dataset used in this study was collected from July 2013 to December 2024, enabling a comprehensive temporal assessment of the dynamics of bacterial blight disease.

Bacterial blight severity was assessed through a structured field survey conducted by trained scouts. In each village, four orchards (two fixed and two randomly selected), each one acre in size, were surveyed. Observation was made from 2090 unique village of 11 districts of Maharashtra. Within each orchard, observations were recorded from ten systematically selected sites following a zigzag sampling approach, with five trees assessed per site, resulting in 50 trees evaluated per orchard. Disease severity was assessed by visually inspecting the leaves, stems, and fruits for characteristic bacterial blight symptoms. Severity was recorded as the average severity grade for stems, along with a combined average severity grade for leaves and fruits. The recorded severity grades (0-5) were converted into percentage disease severity using the mid-point average method. Observations were recorded at weekly at each location. The village-level data were subsequently aggregated to obtain district-level weekly severity values. The final analytical dataset was constructed by pooling the district-level weekly observations across all nine districts, resulting in a unified multi-district spatiotemporal time-series dataset for downstream statistical, time-series, and ML modelling.

Data collection was facilitated through an Information and Communication Technology (ICT)-based pest and disease surveillance system developed to support large-scale pest monitoring under field conditions. The surveillance system followed a three-tier architecture consisting of (i) a mobile application for field-level data entry, (ii) a centralized database for data storage and management, and (iii) a web-based pest reporting and advisory platform. The system was specifically designed to address the challenges associated with pest surveillance and limited internet connectivity in remote agricultural regions.

Weekly meteorological data were collected from the India Meteorological Department (IMD). The study used key weather parameters measured at multiple time intervals (morning, afternoon, evening, and night), and a complete description of the variables and their temporal scales is presented in **Table 1**. The inclusion of a comprehensive set of weather variables strengthened the analysis by capturing the multidimensional environmental influences on disease progression. These data were used for subsequent statistical, time-series, and ML analyses to examine the disease-weather interactions and seasonal disease patterns.

**Table 1:** Meteorological variables used for disease severity modelling.

S.no	Weather variables	Description	Unit	Abbreviation
1.	Wet bulb temperature (morning)	Mean wet bulb temperature recorded in morning (8.30 AM)	°C	WBT_Mor
2.	Wet bulb temperature (afternoon)	Mean wet bulb temperature recorded in afternoon (11.30 AM)	°C	WBT_Aft
3.	Wet bulb temperature (evening)	Mean wet bulb temperature recorded in evening (5.30 PM)	°C	WBT_Evg
4.	Wet bulb temperature (night)	Mean wet bulb temperature recorded in night (11.30 PM)	°C	WBT_Ngt
5.	Minimum temperature	Daily minimum air temperature	°C	T_min
6.	Maximum temperature	Daily maximum air temperature	°C	T_max
7.	Total rainfall	Total rainfall in 24h ending 08:30 IST	mm	RF
8.	Duration of rainfall	Duration of rainfall in 24h ending 08:30 IST	hours	DRNRF
9.	Dry bulb temperature (morning)	Dry bulb temperature recorded in morning (8.30 AM)	°C	DBT_Mor
10.	Dry bulb temperature (afternoon)	Dry bulb temperature recorded in afternoon (11.30 AM)	°C	DBT_Aft
11.	Dry bulb temperature (evening)	Dry bulb temperature recorded in evening (5.30 PM)	°C	DBT_Evg
12.	Dry bulb temperature (night)	Dry bulb temperature recorded in night (11.30 PM)	°C	DBT_Ngt
13.	Dew point temperature (morning)	Mean dew point temperature recorded in morning (8.30 AM)	°C	DPT_Mor
14.	Dew point temperature (afternoon)	Mean dew point temperature recorded in afternoon (11.30 AM)	°C	DPT_Aft
15.	Dew point temperature (evening)	Mean dew point temperature recorded in evening (5.30 PM)	°C	DPT_Evg
16.	Dew point temperature (night)	Mean dew point temperature recorded in night (11.30 PM)	°C	DPT_Ngt
17.	Relative humidity (morning)	Mean relative humidity recorded in morning (8.30 AM)	%	RH_Mor
18.	Relative humidity (afternoon)	Mean relative humidity recorded in afternoon (11.30 AM)	%	RH_Aft
19.	Relative humidity (evening)	Mean relative humidity recorded in evening (5.30 PM)	%	RH_Evg
20.	Relative humidity (night)	Mean relative humidity recorded in night	%	RH_Ngt
21.	Evaporation	Total evaporation in 24h ending 08:30 IST	mm	EVP
22.	Sunshine hours	Duration of sunshine	hours	SSH
23.	Average wind speed	Average wind speed in km/hrs	Kmh^-1^	AW

### 2.2 SMW-based seasonal analysis

Disease severity data from daily observations were averaged to obtain weekly (corresponding to each SMW) and monthly bacterial blight severity. Disease severity observations collected from 2013 to 2024 were summarized on a monthly and SMW basis to evaluate seasonal patterns and identify periods of disease occurrence. Seasonal analysis of bacterial blight severity was performed using both monthly and SMW data. Multiple graphical representations, including monthly distributions, SMW-wise line plots, bar plots, and trend plots, were generated to assess the consistency of seasonal behaviour over the years.

### 2.3 Correlation analysis

Correlation analysis was employed as a statistical approach to examine how variations in bacterial blight severity aligned with changes in key meteorological variables. The Pearson correlation coefficient was used to measure the degree and direction of linear relationships between the disease severity index and selected weather parameters, including temperature (max and min), relative humidity, rainfall, wind speed, sunshine duration, and other relevant atmospheric indicators as provided in **Table 1**.

### 2.4 Regression model

Regression-based modelling was employed to quantify and predict bacterial blight severity using concurrent meteorological data. All statistical analyses and model implementations were performed in R software (version 4.5.2). The dataset consisted of weekly bacterial blight severity observations paired with corresponding weather parameters and was pre-processed prior to model fitting. Predictors with >90% missing values were excluded, and the remaining missing values were handled using median imputation to ensure a complete dataset for subsequent analysis. The regression approaches were classified into (i) statistical regression models and (ii) ML–based regression models:

#### 2.4.1 Statistical regression models

- Linear Regression (LR) and Principal Component Analysis coupled with Linear Regression (PCA–LR): LR was performed using stepwise selection to identify significant predictors, with the *caret* and *dplyr* libraries for model fitting and pre-processing. Similarly, PCA-LR was applied to reduce predictor dimensionality while retaining 95% variance, followed by LR on the resulting principal components using the *caret* library with bootstrapped resampling. The model performance for both approaches was evaluated using the Root Mean Square Error (RMSE), Mean Absolute Error (MAE), and coefficient of determination (R²).
- Least Absolute Shrinkage and Selection Operator (LASSO) regression: Implemented using the *glmnet* function with 10-fold cross-validation to identify the optimal regularization parameter (λ) that minimizes the prediction error. Both the minimum λ (lambda.min) and a more conservative λ (lambda.1se) were considered to balance model complexity and generalization. Model performance was evaluated using RMSE, R², and MAE, while variable importance was determined from the absolute magnitude of the coefficients at lambda.min.

#### 2.4.2 ML-based regression models

Random Forest (RF): RF Regression was implemented using the *randomForest* package, with data pre-processing handled via *dplyr* and visualization using *ggplot2*. The model was trained with 500 trees (ntree), considering seven variables at each split to allow the algorithm to be optimized based on the data. The variable importance was quantified using the percentage increase in the mean squared error (%IncMSE).

Support Vector Regression (SVR): Radial kernel with type eps-regression, with cost (10) and gamma (0.1) parameters were set to control model flexibility and non-linearity. The model performance was evaluated using the RMSE, R², and MAE, and the number of support vectors was examined to assess the model complexity.

Generalized Additive Models (GAM): GAM was used to capture non-linear relationships between selected meteorological variables and disease severity. Smooth functions were fitted to the predictors, and the significance of each term was assessed. Model performance was summarized using adjusted the R², GCV, and scale estimates.

Light Gradient Boosting Machine (LightGBM): LightGBM regression was implemented using the *lightgbm* package, employing a gradient-boosted decision tree framework with a regression objective function. Model performance was evaluated using RMSE and the R². The model was trained using 600 trees with a learning rate of 0.05. Tree complexity and generalization were controlled through 31 leaves per tree, feature subsampling of 0.8, bagging fraction of 0.8, and bagging frequency of 5.

Extreme Gradient Boosting (XGBoost): XGBoost regression was performed using the *xgboost* package and trained by a gradient-boosted tree framework with a squared error objective function over 500 boosting iterations. The key hyperparameters included a learning rate of 0.05, maximum tree depth of 6, subsampling ratio of 0.8, and column subsampling ratio of 0.8.

To further enhance the predictive performance, a Bayesian-optimized XGBoost model was developed using the *ParBayesianOptimization* framework. Hyperparameter tuning was conducted through five-fold cross-validation by optimizing the learning rate (0.01, 0.3), maximum tree depth (3, 10), subsampling ratio (0.5, 1.0), and column subsampling ratio (0.5, 1.0). The optimal configuration identified a learning rate of 0.023, maximum depth of 7, subsampling ratio of 0.684, and a column-subsampling ratio of 0.606. Except for the Bayesian-optimized XGBoost model, the other ML models were not subjected to extensive hyperparameter tuning, therefore; the comparative analysis was intended to provide exploratory insights rather than a rigorous benchmarking of model performance. A concise summary of key hyperparameters and their configurations for all ML models is provided in **Table 2**. In addition, for both the standard and tuned XGBoost models, SHapley Additive exPlanations (SHAP) analysis was performed to interpret the contribution of individual features to the model predictions.

**Table 2:** Summary of the key hyperparameters and their configurations for the ML models.

Model	Key hyperparameters	Configuration	Tuning Status
Random Forest (RF)	ntree, mtry	ntree = 500; mtry = 7	Default
Support Vector Regression (SVR)	kernel, cost, gamma	radial kernel; cost = 10; gamma = 0.1	Default
Generalized Additive Model (GAM)	smoothing functions	smooth terms fitted	Default
LightGBM	num_trees, learning_rate, num_leaves, feature_fraction, bagging_fraction	600 trees; lr = 0.05; leaves = 31; feature_fraction = 0.8; bagging_fraction = 0.8	Default
XGBoost (baseline)	nrounds, learning_rate, max_depth, subsample, colsample_bytree	500 rounds; lr = 0.05; depth = 6; subsample = 0.8; colsample = 0.8	Default
XGBoost (Bayesian optimized)	learning_rate, max_depth, subsample, colsample_bytree	lr = 0.023; depth = 7; subsample = 0.684; colsample = 0.606	Tuned (Bayesian optimization)

#### 2.4.3 Model evaluation and comparative assessment

The predictive performance of all regression models, including both statistical and ML-based models, was evaluated using standard accuracy metrics, including the RMSE and R². In addition, a comparative evaluation of the ML models (RF, SVR, XGBoost, and LightGBM) was performed using a radar plot to visualize the relative predictive performance across the evaluation metrics.

For hyperparameter tuning and model selection during training, an expanding window time-series cross-validation (TSCV) was implemented using the ‘tsCV’ function from the ‘forecast’ package. The procedure used 3 folds with an initial training window of 200 weeks, expanding by 50 weeks each iteration. This approach ensures that validation observations always occur after training observations, preventing look-ahead bias.

### 2.5 Time-series forecasting algorithm

Time-series analysis was applied to evaluate the temporal continuity, seasonal behaviour, and dynamic patterns of bacterial blight severity. Analyses were performed on data aggregated at the SMW level to ensure temporal alignment and support seasonal modelling. Multiple time-series approaches were employed, including ARIMA and SARIMA models for trend and seasonal pattern analyses, and VAR modelling to assess the dynamic interrelationships among variables.

#### 2.5.1 ARIMA

The Autoregressive Integrated Moving Average (ARIMA) model was employed to analyse temporal variation and forecast bacterial blight severity in pomegranate cultivation. The ARIMA framework combines three components: the autoregressive (AR) term, which captures dependence on previous observations; the integrated (I) term, which represents the differencing applied to achieve stationarity; and the moving average (MA) term, which accounts for serial correlation in the error. The stationarity of the series was examined using the ADF test to determine the need for differences. Model selection was performed using an automated ARIMA approach, in which the optimal model order (p, d, and q) was identified based on the minimum Akaike Information Criterion (AIC), corrected AIC (AICc), and Bayesian Information Criterion (BIC) values.

The selected ARIMA model was fitted to the weekly severity series, and the fitted values were obtained to assess temporal trends. The predictive performance of the model was quantitatively evaluated using standard accuracy metrics, including RMSE, MAE, Mean Absolute Percentage Error (MAPE), and R². Model adequacy and residual independence were assessed using a series of diagnostic tests. The Autocorrelation Function (ACF) and Partial Autocorrelation Function (PACF) plots of the residuals were examined to detect any remaining serial dependence. Similarly, the Ljung–Box Q test and residual normality were assessed using the Jarque–Bera and Shapiro–Wilk tests.

#### 2.5.2 SARIMA

Seasonal Autoregressive Integrated Moving Average (SARIMA) modelling was applied to evaluate potential seasonal variations in weekly bacterial blight severity. Model selection was performed using an automated procedure (*auto.arima*), which identifies the optimal model parameters by considering both non-seasonal and seasonal autoregressive and moving average components across SMWs. The model selection was based on the minimum AIC, AICc, and BIC values. Model performance was evaluated using the RMSE, MAE, MAPE, and R². The ACF and PACF plots of the residuals were examined to detect any remaining serial dependencies. The model adequacy was assessed using residual diagnostics, including the Ljung–Box Q test for residual autocorrelation, Jarque–Bera and Shapiro–Wilk tests for normality, and the ADF test for stationarity.

#### 2.5.3 VAR modelling

The VAR model was applied as a multivariate time-series framework to jointly analyse the bacterial blight severity and associated meteorological variables, such as temperature, relative humidity, and rainfall. Prior to model fitting, the stationarity of each time-series was examined using the ADF test, and the remaining missing values were handled using linear median interpolation to maintain temporal continuity.

The model adequacy was evaluated using comprehensive residual diagnostics. Serial autocorrelation was examined using the Ljung–Box Q and Portmanteau tests, which assess whether residual autocorrelation exists across multiple lags, whereas heteroscedasticity was assessed using the Autoregressive Conditional Heteroskedasticity (ARCH) test. Residual normality was evaluated using the Jarque–Bera and Shapiro–Wilk tests, and model stability was examined using the cumulative sum (CUSUM) test. Dynamic interactions among variables were further explored using Impulse Response Functions (IRF) and Forecast Error Variance Decomposition (FEVD), and Granger causality analysis was applied to assess the directional relationships. The model performance was evaluated using RMSE, MAE, MAPE, and R², and the predictive performance was compared with that of the univariate ARIMA and SARIMA models.

## 3 Results

### 3.1 SMW based seasonal analysis

**Figure 2:**
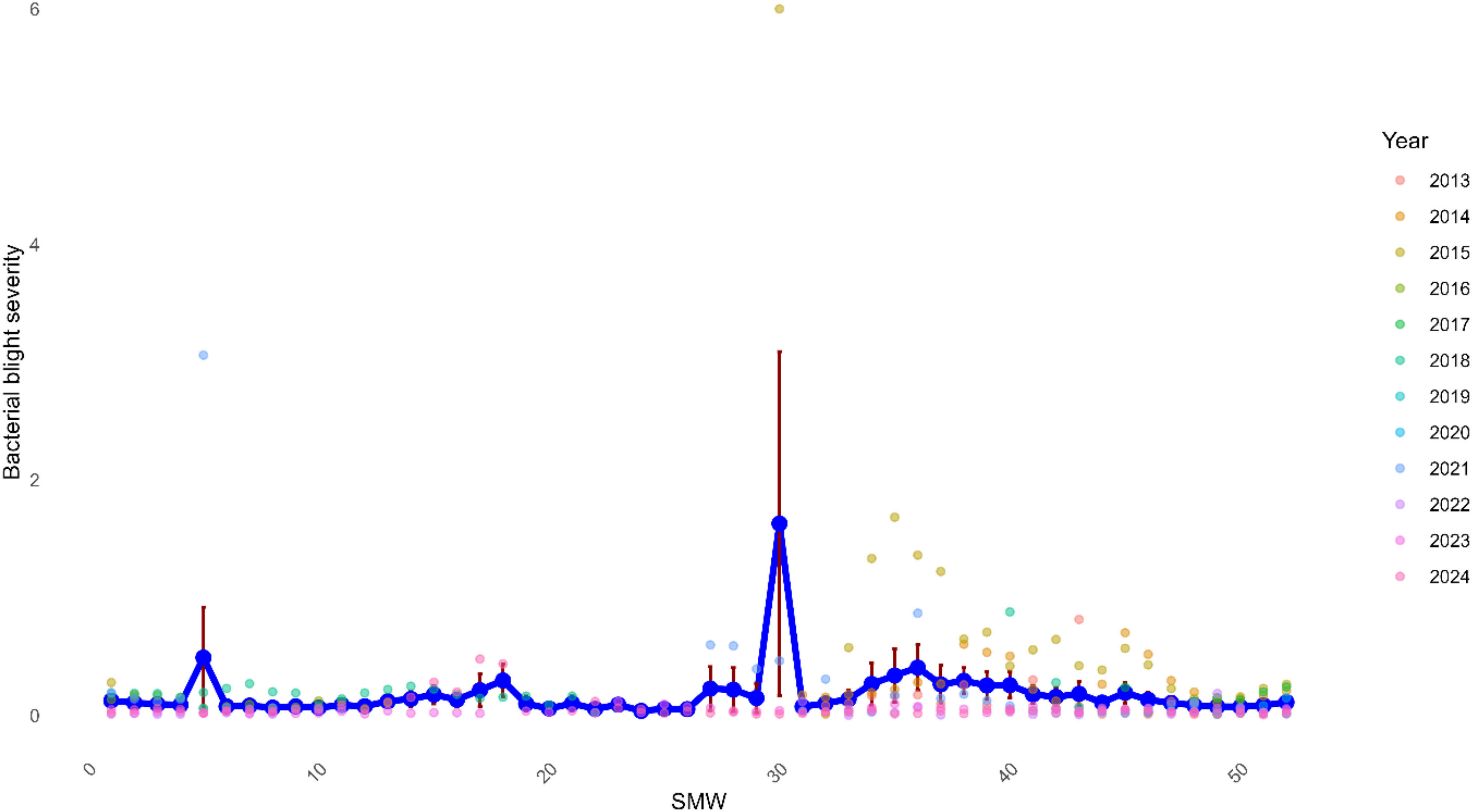
SMW-wise distribution of mean bacterial blight severity across individual years from 2013 to 2024.

Seasonal analysis based on SMWs demonstrated a distinct temporal pattern in bacterial blight severity from 2013-2024. The SMW-wise severity trend (**Figure 2**) showed a sharp and consistent peak during SMW 29–31, corresponding to July, indicating the primary occurrence of bacterial blight. Year-wise SMW analysis further revealed pronounced interannual variation, with 2015 recording the highest disease severity, characterized by a prominent peak centered at SMW 30. During this peak week, the mean bacterial blight severity was 0.5, which was higher than the severity levels observed during the same SMWs in other years. In contrast, disease severity remained low and stable across the remaining SMWs, confirming a narrow and well-defined seasonal outbreak.

**Figure 3:**
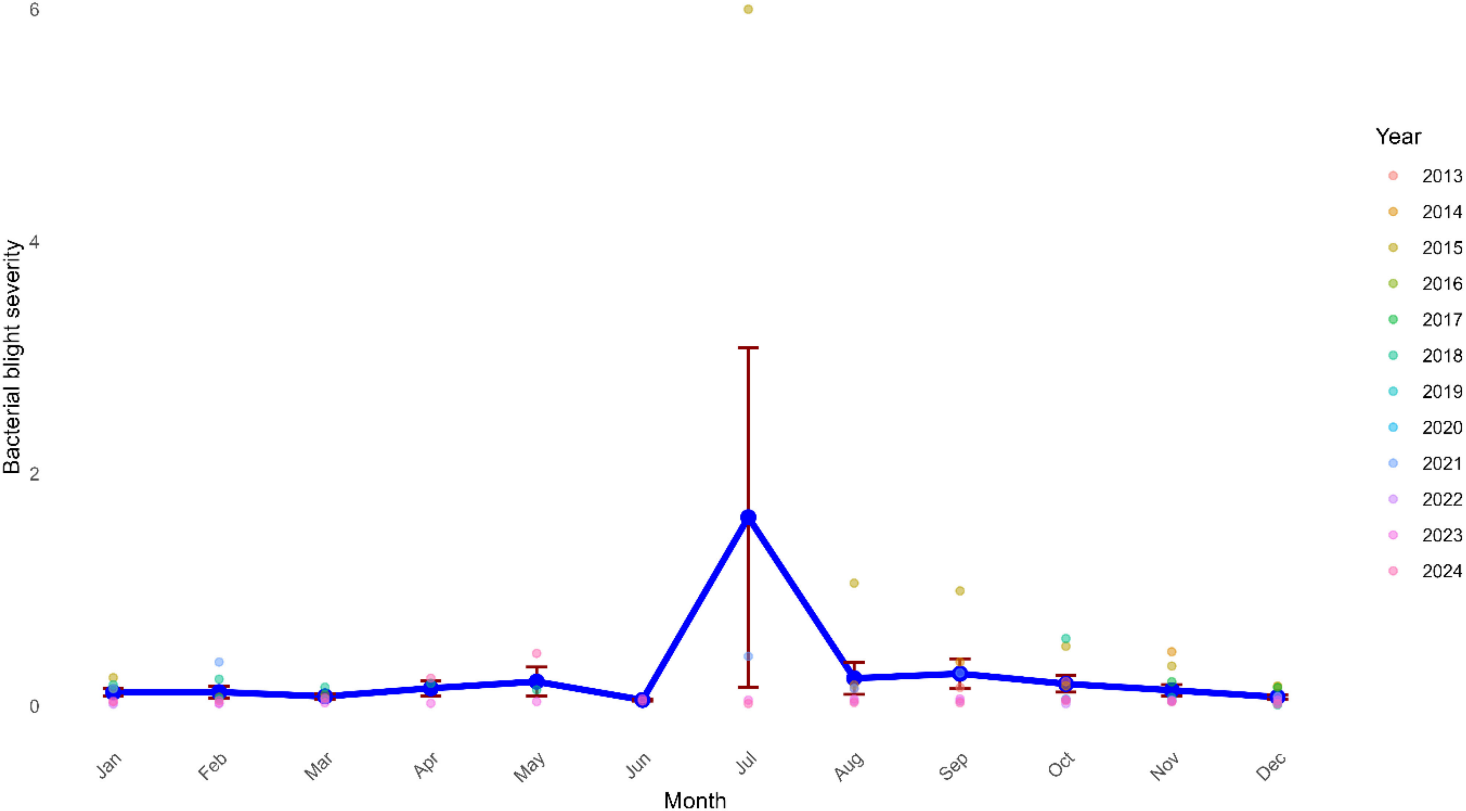
Monthly mean bacterial blight severity across 2013-2024, highlighting July as the primary month of peak disease occurrence.

Monthly analysis further supported the SMW-based findings by identifying July as the most critical month for bacterial blight development (**Figure 3**). The year-specific monthly severity trend demonstrated that July 2015 recorded the maximum monthly bacterial blight severity (mean severity = 6), reflecting the most severe outbreak observed during the study period. When monthly severity values were averaged across all years (2013–2024), July exhibited the highest long-term mean bacterial blight severity at 1.62, whereas other months showed comparatively lower mean severity values, highlighting a strong association between mid-monsoon conditions and disease escalation. In addition, all graphical representations produced consistent seasonal trends, as shown in **Supplementary Figures S1**-**S3**.

### 3.2 Correlation analysis

**Figure 4:**
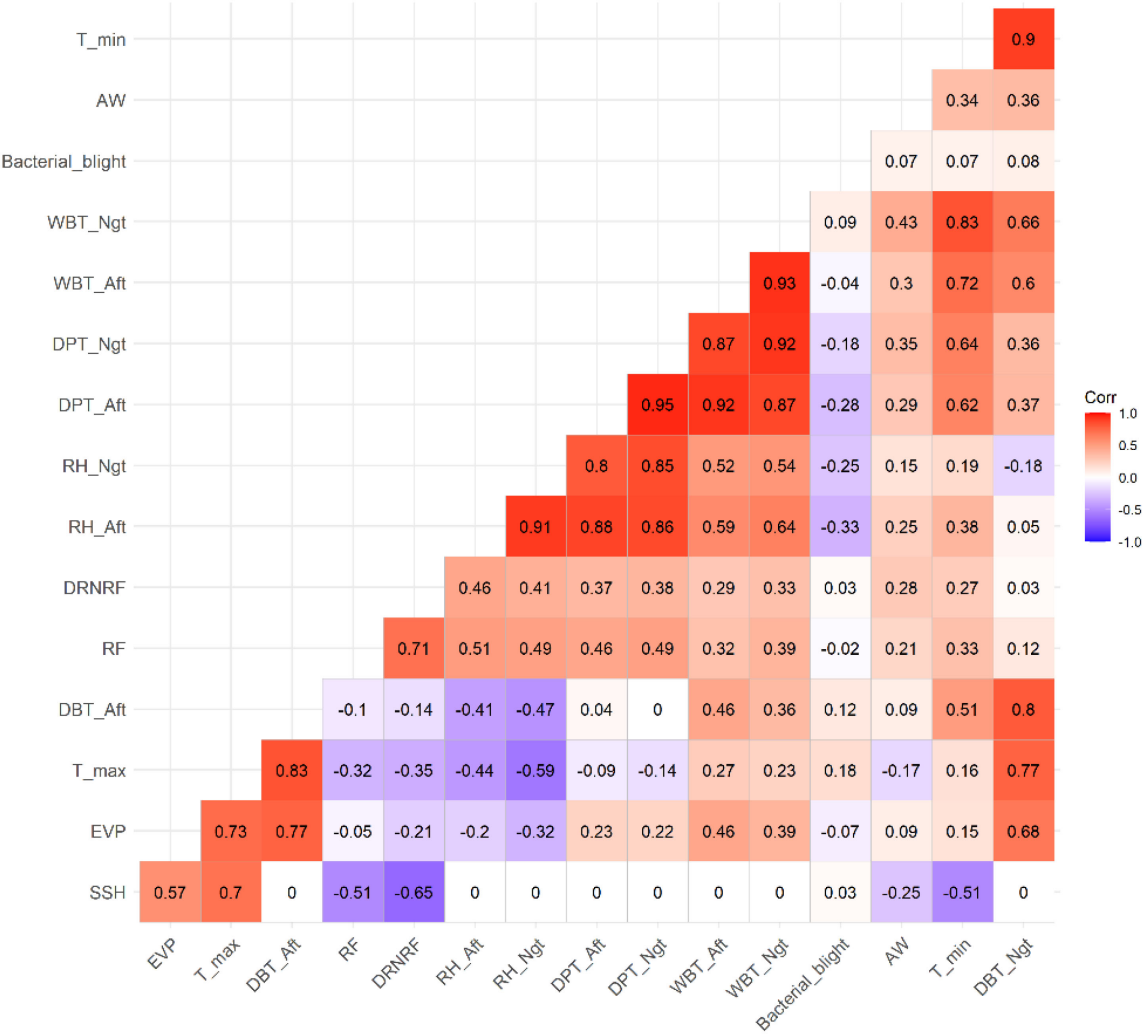
The correlation matrix represents the Pearson correlation coefficient between bacterial blight severity and the meteorological variables.

Correlation analysis indicated that bacterial blight severity exhibited weak negative and weak positive associations with most of the examined meteorological parameters (**Figure 4**). Among the negatively correlated parameters, bacterial blight severity showed weak inverse relationships with afternoon RH (r = −0.33), indicating that an increase in RH was associated with a decrease in disease severity. DPT in the afternoon also demonstrated weak negative correlations (r = −0.28), suggesting a similar declining trend in bacterial blight severity with increasing DPT. These results collectively indicate that these meteorological parameters exert a weak suppressive effect on the severity of bacterial blight.

In contrast, weak positive correlations were observed between the bacterial blight severity and temperature. Maximum temperature showed the maximum positive correlation with disease severity (r = 0.18), followed by DBT in afternoon (r = 0.12), indicating a slight increase in disease severity with increasing temperature. Rainfall (r = −0.02) and DRNRF (r = 0.03) exhibited negligible relationships with the disease incidence. Overall, the correlation analysis revealed that bacterial blight severity from field-level data in pomegranates was weakly influenced by meteorological parameters, as reflected by the low correlation magnitudes, suggesting limited linear dependence between disease severity and individual weather variables.

### 3.3 Regression model

#### 3.3.1 Statistical regression model

The predictive performance on held out testing data of both statistical and ML regression models was evaluated using RMSE and R², as summarized in **Table 3**; **Supplementary Figure S4**. All statistical regression models demonstrated varying predictive performances in estimating bacterial blight severity. The linear regression model showed an RMSE of 1.011, an R² value of 0.286, and an MAE of 0.802, indicating moderate predictive accuracy. PCA-linear had the lowest predictive performance, with an RMSE of 1.137, an R² value of 0.094, and an MAE of 0.907, while retaining 95% of the total variance. The GAM further improved the model representation of non-linearity, with an adjusted R² value of 0.33, a GCV value of 0.988, and a scale estimate of 0.961, indicating a stable model fit for bacterial blight severity prediction.

**Table 3:** Comparative performance of all regression models in predicting bacterial blight severity on testing data.

Model	RMSE	R^2^
Linear regression (LR)	1.011	0.286
LASSO	1.001	0.300
PCA-linear	1.136	0.099
LightGBM	0.566	0.776
Random forest (RF)	0.584	0.762
SVR	0.700	0.657
XGBoost	1.560	0.698
Bayesian optimized XGBoost	0.823	0.536

**Figure 5:**
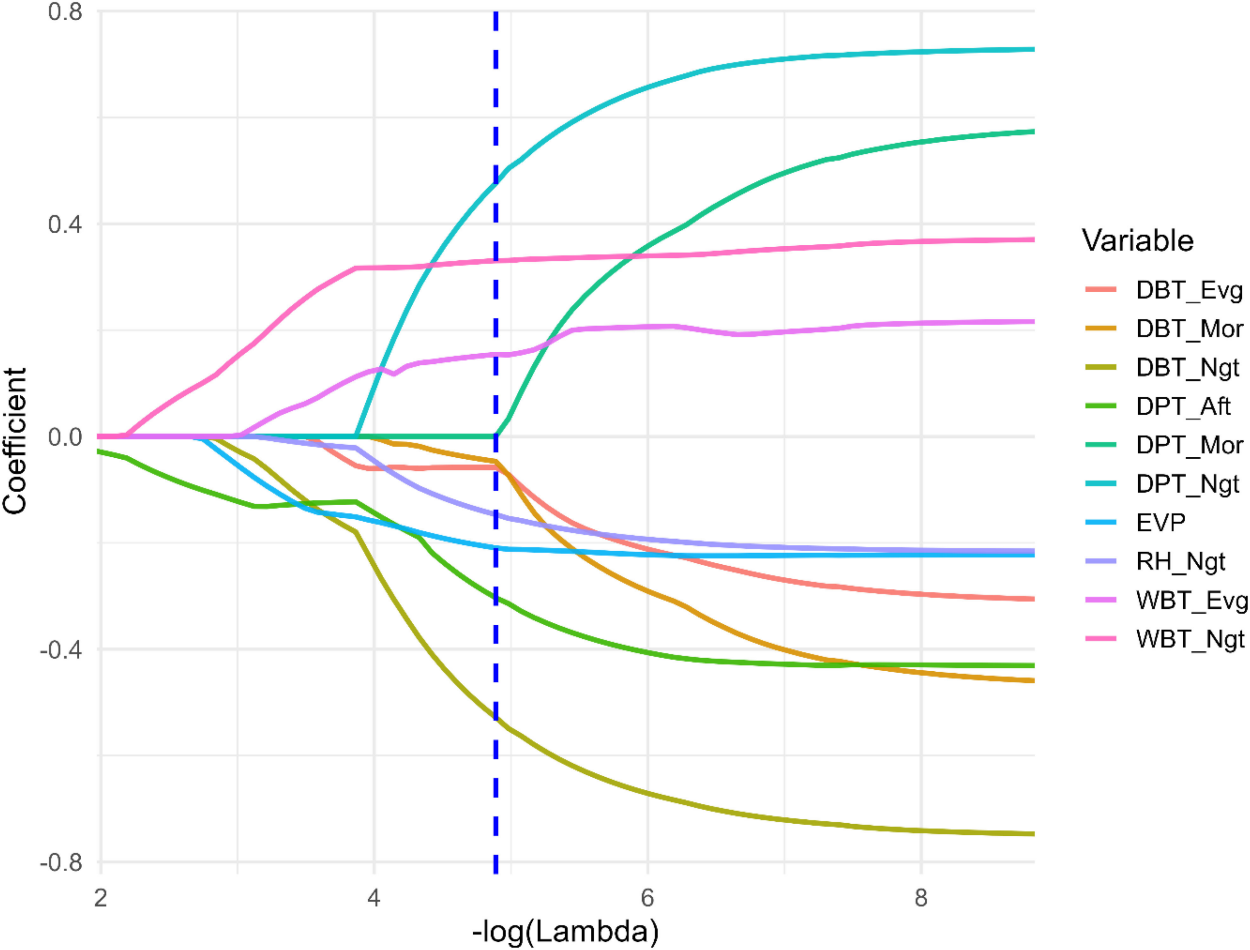
LASSO coefficient paths for meteorological variables associated with bacterial blight severity.

LASSO regression enabled weightage-based identification of key meteorological drivers influencing bacterial blight severity shown in **Figure 5**. In the optimal regularization parameter (λ_min = 0.00013), the model achieved an R² value of 0.300. At the more regularized setting (λ_1se = 0.00754), a reduced set of predictors was retained with stable predictive performance. Weightage analysis revealed that DBT_Ngt (0.747) and DPT_Ngt (0.728) were the most influential parameters, indicating a strong contribution to disease severity. Moderate influence was observed for EVP (0.222) and DBT_Evg (0.306), while RF (0.026) exhibited the lowest weight, indicating a minimal contribution.

#### 3.3.2 ML-based regression models

Among all ML-based regression models, LightGBM demonstrated a strong predictive performance on the held-out test data, for bacterial blight severity, yielding an RMSE of 0.566 and an R² value of 0.776. SVR demonstrated superior performance compared to statistical models, achieving an RMSE of 0.700 and an R² value of 0.657, reflecting improved capture of non-linear relationships between meteorological variables and disease severity. The RF model achieved an RMSE of 0.584, an R² value of 0.762, and a mean squared residual (MSR) of 0.562. In addition to the overall prediction accuracy, RF provides an assessment of predictor importance based on the percentage increase in mean squared error (%IncMSE). As shown in **Supplementary Figure S5**, T_min emerged as the most influential predictor, with a %IncMSE of 59.662%, followed by AW (53.712%) and T_max (49.089%). Rainfall and evaporation also contributed substantially to the model performance. In contrast, RH_Mor showed the lowest importance, with an %IncMSE of 15.396%, indicating a comparatively weaker influence on the bacterial blight severity prediction. Overall, the RF importance analysis highlighted the dominant role of temperature in shaping the patterns of disease severity.

**Figure 6:**
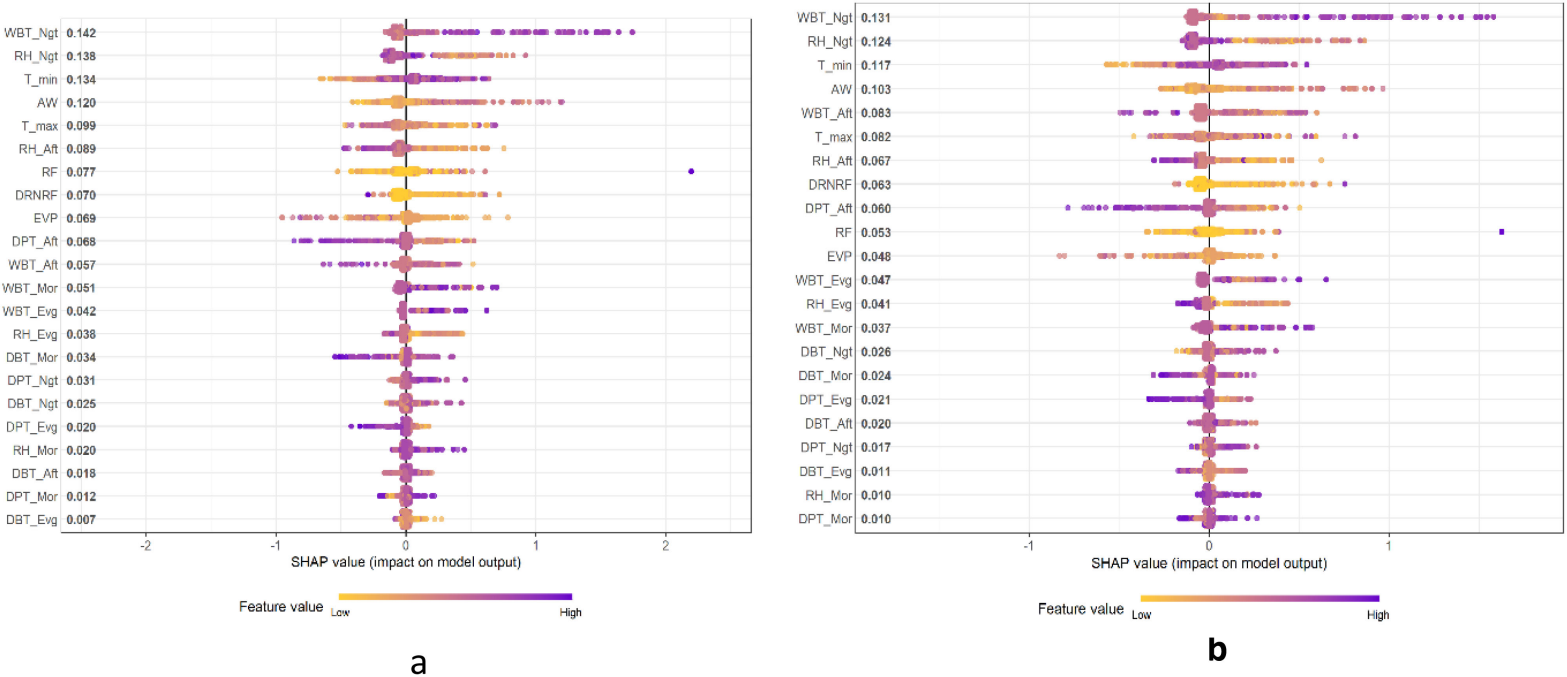
**(a)** SHAP summary of XGBoost, **(b)** SHAP summary of Bayesian-optimized XGBoost models.

The baseline XGBoost regression model showed limited predictive performance with an RMSE of 1.560, while Bayesian optimization XGBoost substantially improved model accuracy, with an RMSE of 0.823. To further interpret the model behaviour, SHAP analysis was performed for both the standard and Bayesian-optimized XGBoost models to identify the most influential meteorological predictors in both the baseline and optimized models (**Figure 6**). In the baseline XGBoost model, WBT_Ngt exhibited the highest mean SHAP value (0.142), followed by RH_Ngt (0.138) and T_min (0.134), indicating their dominant contribution to bacterial blight severity prediction, whereas DBT_Evg showed the lowest influence (0.007). A consistent ranking was observed in the Bayesian-optimized XGBoost model, where WBT_Ngt remained the most influential variable (SHAP = 0.131), followed by RH_Ngt (0.124) and T_min (0.117), while RH_Mor and DPT_Mor contributed minimally (0.010). The similarity in the SHAP value distributions across both models confirmed the stability of the key meteorological drivers influencing bacterial blight severity.

Among the ML models, LightGBM emerged as the strongest performing model, achieving the lowest RMSE (0.566), indicating superior prediction of bacterial blight severity. XGBoost showed the lowest predictive performance, as reflected by its higher RMSE (1.560). Considering all statistical and ML models, LightGBM consistently emerged as the best-performing model, demonstrating the highest overall predictive accuracy for bacterial blight severity.

**Figure 7:**
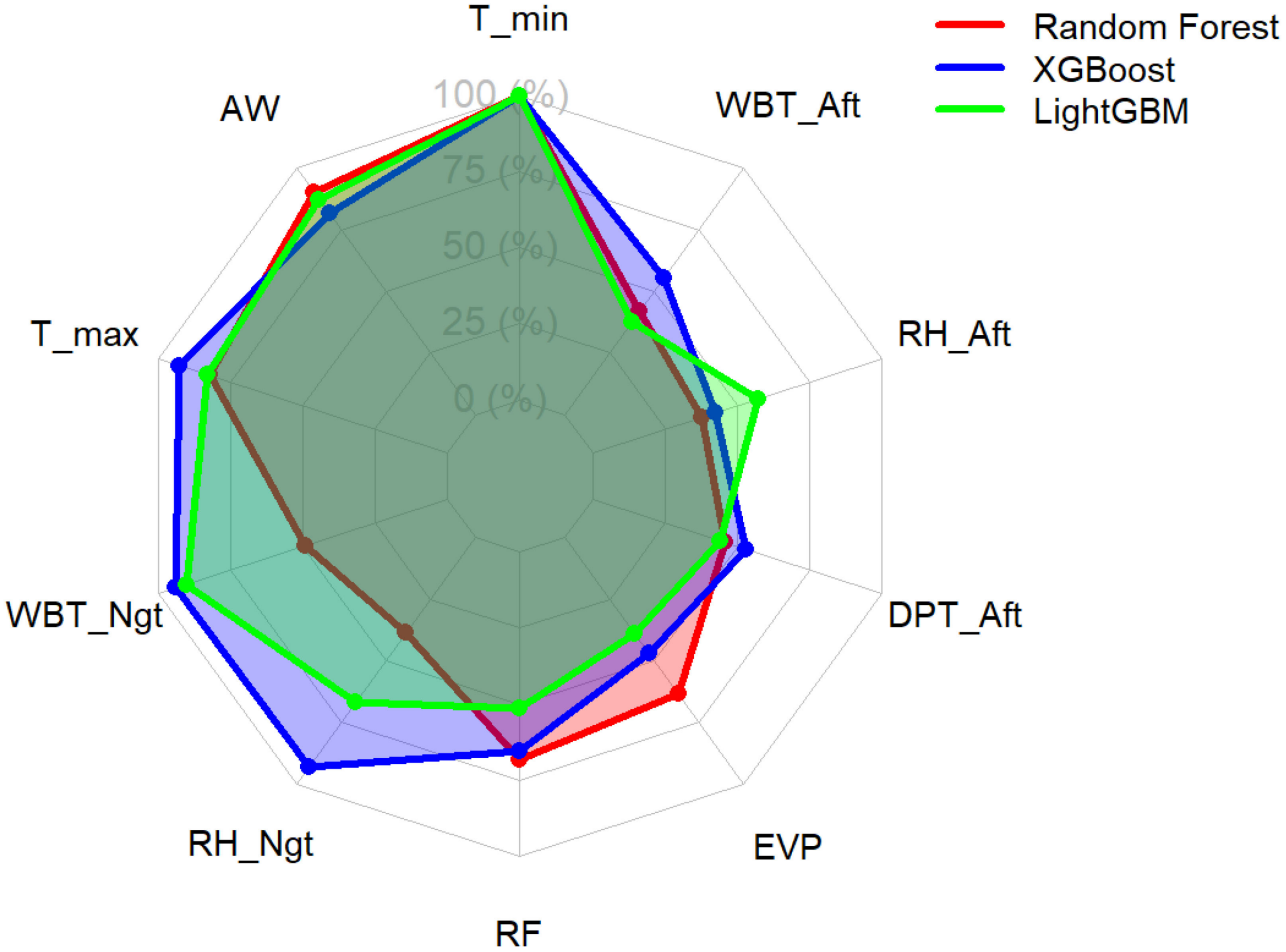
Radar plot showing the comparative variable importance across the different ML models.

A radar plot was used to compare the relative importance of the top meteorological predictors across the RF, XGBoost, and LightGBM models (**Figure 7**). The plot illustrates that different algorithms prioritize key climatic variables, with T_max consistently showing the highest contribution across all models. RF emphasized evaporation and rainfall, indicating differences in sensitivity to short-term and delayed environmental effects. LightGBM exhibited a more evenly distributed importance pattern across all the predictors. XGBoost assigned the highest frequency to key weather parameters, indicating their strong contribution to the prediction of bacterial blight severity. Among all predictors, T_max showed the highest frequency (0.2247), followed by T_min (0.1596), indicating that temperature was the most consistently used predictor by the models during tree splitting. WBT_Ngt (0.0224) and RH_Ngt (0.0235) showed moderate importance, whereas DPT_Mor and RH_Evg displayed comparatively lower frequencies (<0.02). Overall, the radar plot highlights model-specific feature attribution rather than predictive accuracy, demonstrating how different ML approaches interpret the drivers of bacterial blight severity.

### 3.4 Time-series analysis

#### 3.4.1 ARIMA model

Automated model selection identified ARIMA (p=1, d=0, and q=0) with a non-zero mean as the optimal model for weekly bacterial blight severity, based on the lowest AIC (102.39), AICc (101.89), and BIC (96.53) values. The autoregressive parameter (AR_1_ = 0.4305) indicated short-term temporal dependence, suggesting that disease severity in a given week was moderately influenced by the severity of the previous week.

**Figure 8:**
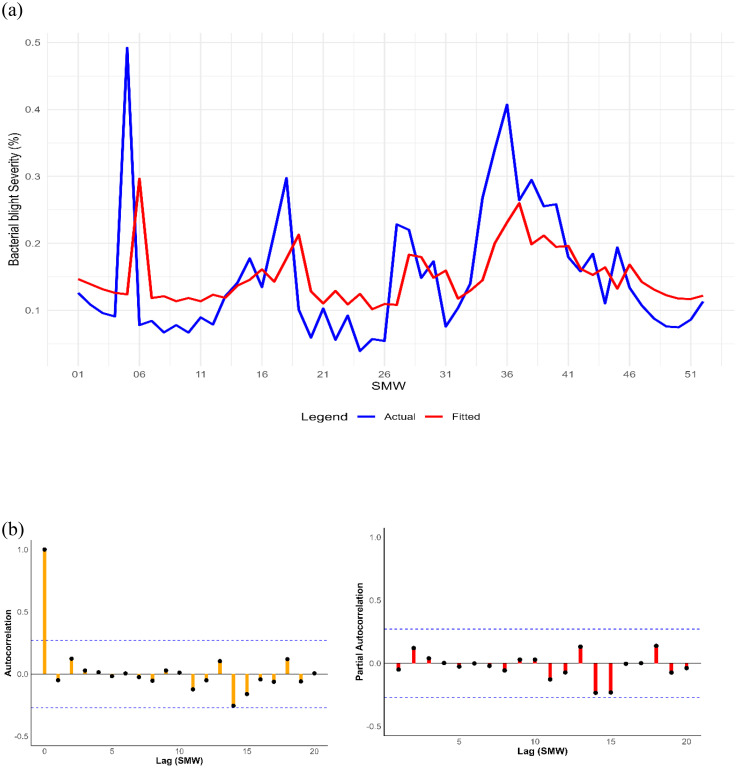
**(a)** ARIMA model trend plot showing observed (actual) and model-predicted (fitted) bacterial blight severity (%) across SMWs during the study period from to 2013-2024. **(b)** Autocorrelation (ACF) and partial autocorrelation (PACF) plots of bacterial blight severity in pomegranate residuals across SMW lags.

The ARIMA trend plot illustrating observed and fitted values showed that the model reasonably captured the temporal variation in bacterial blight severity across SMWs shown in (**Figure 8A**). Bacterial blight severity during 2013–2024 ranged from approximately 0.05% to 0.50%, with a distinct early season peak during SMW 5–6 (~0.50%), followed by reduced severity during the mid-season (SMW 24–26). A secondary increase was observed during SMW 35–37 (~0.40%), indicating a late-season resurgence. The fitted series closely followed the observed pattern, particularly during periods of low-to-moderate severity. The model performance metrics indicated an RMSE of 0.085, MAE of 0.058, MAPE of 48.48%, and R² value of 0.691, reflecting moderate predictive accuracy in representing weekly disease dynamics.

Residual independence was confirmed using the Ljung–Box Q test, which showed a non-significant result (p = 0.914), indicating the absence of residual autocorrelation and confirming that temporal dependence was adequately addressed by the ARIMA model. The ACF plot showed that residual correlations remained largely within the confidence limits across SMW lags, while the PACF plot did not exhibit pronounced spikes at early lags, suggesting a lack of a dominant autoregressive structure in the residuals (**Figure 8B**). Normality and stationarity diagnostics did not indicated deviations from the ideal assumptions.

#### 3.4.2 SARIMA

Automated model selection identified the SARIMA model with non-seasonal parameters (p=2, d=1, and q=1) as the optimal model for weekly bacterial blight severity based on the lowest information criterion values (AIC = 11.68, AICc = 11.75, and BIC = 29.21). The estimated autoregressive parameters indicate short-term temporal dependence, whereas the moving average component captures the residual variability in the series. Notably, the seasonal components were not retained in the final model.

**Figure 9:**
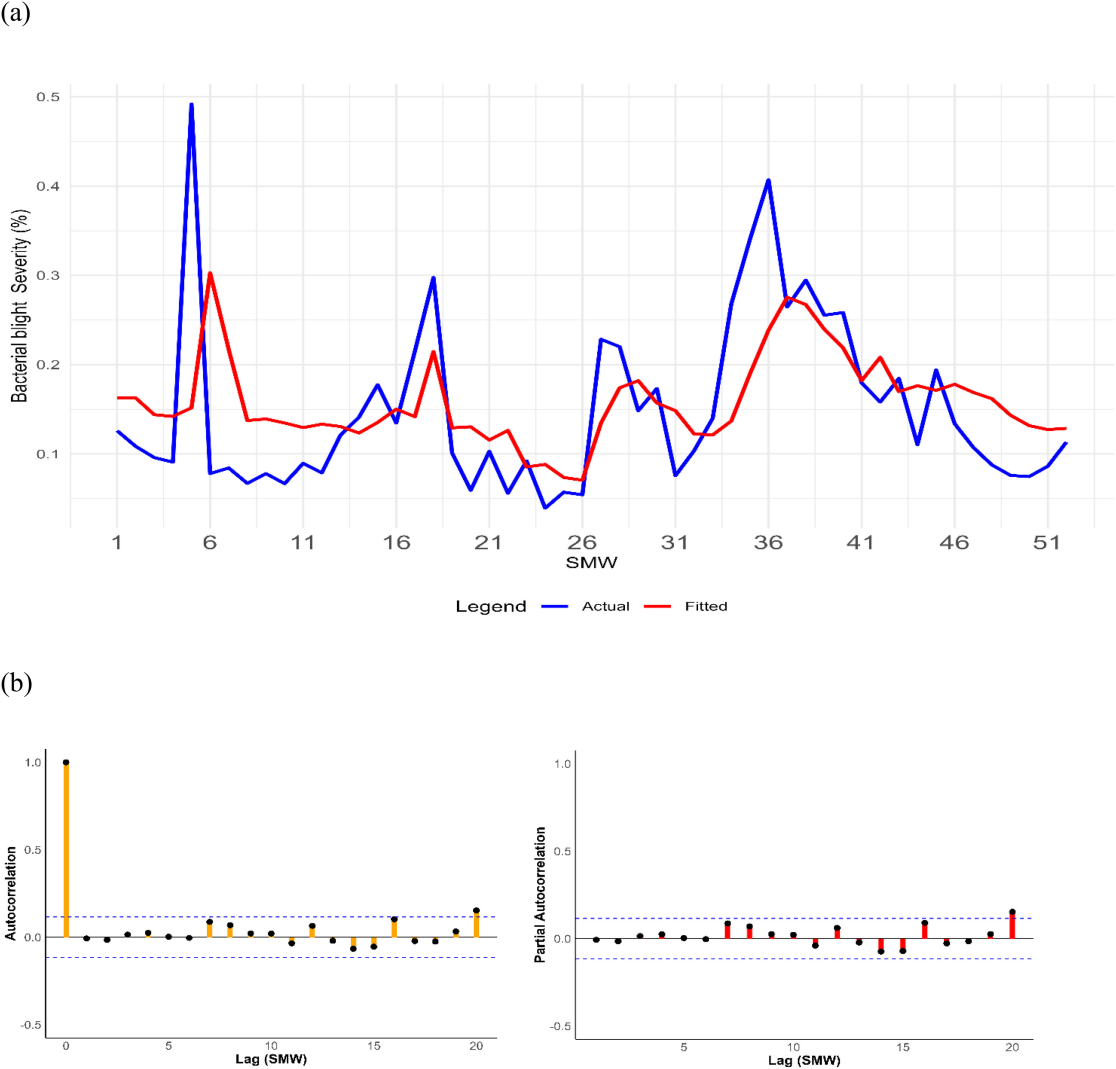
**(a)** SARIMA model trend plot showing observed (actual) and model-predicted (fitted) bacterial blight severity (%) across SMWs. **(b)** Autocorrelation (ACF) and partial autocorrelation (PACF) plots of bacterial blight severity in pomegranate residuals across SMW lags.

The SARIMA-fitted values closely followed the observed severity pattern across the SMW, effectively capturing the temporal fluctuations in bacterial blight severity (**Figure 9A**). Model accuracy metrics indicated an RMSE of 0.24, MAE of 0.103, and an R² value of 0.758, reflecting a satisfactory model fit and predictive performance for disease severity. The averaging of the fitted values further demonstrated a consistent alignment between the observed and predicted severity trends across SMWs.

The residual diagnostics supported the adequacy of the SARIMA model. A non-significant Ljung–Box Q test (p = 0.489) confirmed the absence of residual autocorrelation, demonstrating an adequate model fit for the data. The ACF and PACF plots of the residuals did not exhibit pronounced spikes across SMW lags, further confirming the absence of a remaining serial correlation (**Figure 9B**).

#### 3.4.3 VAR

The VAR model was applied as a multivariate time-series approach to jointly analyse bacterial blight severity along with associated weather parameters, allowing disease dynamics to be interpreted in relation to concurrent meteorological conditions rather than relying solely on past values. The stationarity of all variables was confirmed using the ADF test for bacterial blight severity and all weather variables (p < 0.05), indicating that the series were suitable for VAR modelling. The dynamic interrelationships between bacterial blight severity and meteorological variables were further explored using IRF, FEVD, and Granger causality testing. IRF revealed that a shock to temperature induced a gradual and delayed response in bacterial blight severity within the 95% bootstrap confidence interval, indicating a lagged effect of temperature on disease dynamics. FEVD results (**Supplementary Figure S5**) demonstrated that bacterial blight severity accounted for approximately 95–100% of its forecast error variance across all forecast horizons, indicating the strong persistence and dominance of intrinsic disease dynamics. Temperature and humidity contributed marginally (<0.01) at longer horizons, while rainfall exhibited a negligible contribution, implying that meteorological variables modulate disease severity indirectly rather than acting as dominant drivers. Granger causality analysis further confirmed the statistically significant causal effects of weather variables on bacterial blight severity.

**Figure 10:**
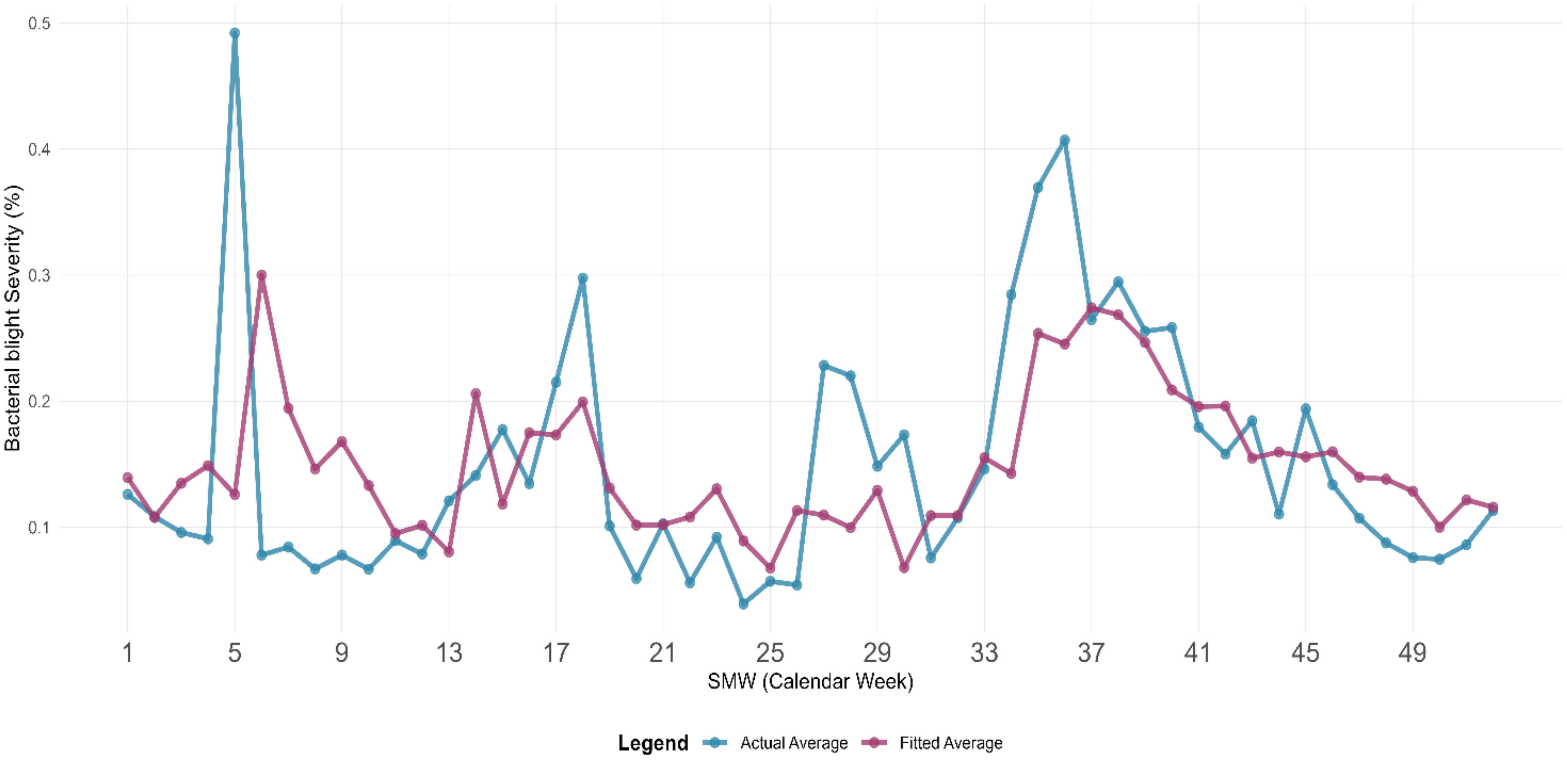
SMW-wise comparison of observed and VAR model–fitted bacterial blight severity averaged across the study period (2013–2024).

The SMW-wise averaged actual versus fitted values further demonstrated that the VAR model captured the seasonal pattern and major peaks of bacterial blight severity reasonably well, particularly during periods of elevated disease pressure, although minor deviations were observed during abrupt fluctuations (**Figure 10**). Based on the model accuracy matrix, the VAR model yielded an RMSE of 0.2398, MAE of 0.1132, and an R² of 0.732, indicating a moderate relationship between the observed and fitted values at the observation level. Additionally, performance evaluation based on SMW-wise averaged actual versus fitted values revealed improved alignment with lower error magnitudes (RMSE of 0.0848, MAE of 0.0580).

## 4 Discussion

This study analysed long-term field collected surveillance data from 2013 to 2024 with meteorological variables to examine the dynamics of bacterial blight severity in pomegranates across major growing districts in Maharashtra. By combining statistical analyses, time-series forecasting (ARIMA, SARIMA, and VAR), and regression models (statistical and ML), the analyses provide valuable insights into the influence of climatic factors on the disease progression. These findings provide a foundation for developing predictive models and early warning systems to support timely climate-adaptive management strategies for sustainable pomegranate cultivation.

The SMW-based seasonal assessment revealed a prominent peak in bacterial blight severity around the 30^th^ SMW (July), identifying the primary epidemic window. Year-wise analysis indicated that 2015 exhibited the highest disease severity, reflecting a notable inter-annual variation. These observations are consistent with previous studies that reported increasing disease severity from June to July, reaching a maximum by September owing to favourable temperature and humidity conditions (Gowda et al., 2020). In addition, Amin et al. (2017) reported a significant increase in bacterial blight severity in cluster beans during the 3^rd^ and 4^th^ weeks of July, further supporting the critical role of mid-season climatic conditions in disease escalation. The pronounced disease peak in July coincides with intensified monsoon conditions, where high humidity and prolonged canopy wetness create a conducive microenvironment for bacterial proliferation, facilitating host penetration and rapid colonization (Suchita et al., 2023). Overall, the SMW analysis underscores the critical periods of bacterial blight emergence, highlighting the need for preventive measures and timely management strategies to mitigate the disease outbreaks.

Correlation analysis revealed generally weak associations between bacterial blight severity and meteorological variables, indicating a limited linear dependence. Notably, RH exhibited a comparatively stronger inverse relationship with disease severity, indicating that higher humid conditions were associated with reduced disease incidence under the observed field conditions. In contrast, maximum temperature showed a weak positive and highest correlation among positively associated parameters, indicating only a marginal contribution to disease variability. These findings suggest that the independent effects of temperature are limited relative to moisture-related variables. These observations align with prior studies, where Bharti et al. (2023) reported rapid disease escalation during the 4th week of July, with T_max and RH positively correlated with bacterial blight severity, while T_min showed a negative association. Similarly, Haque et al. (2022) observed that disease prevalence increased under high RH (70–84%) and RF (>200 mm), whereas T_max was positively correlated with certain growth stages. Rehman and Atiq (2024), studying rice bacterial blight, documented negative correlations with T_max and T_min and positive associations with RF, RH, and AW. Rashid and Aslam Khan (2017) reported temperature and RH as the dominant driver of bacterial blight severity in cotton, with RF and AW showing weaker relationships. Collectively, these findings are consistent with those of the present study, indicating that bacterial blight severity is largely governed by temperature and humidity interactions, with moisture availability enhancing disease development.

ML regression models, including Random Forest, LightGBM, and Bayesian-optimized XGBoost, were employed to predict bacterial blight severity and capture complex nonlinear interactions between meteorological variables and disease progression. Among the evaluated algorithms, LightGBM demonstrated superior predictive performance, reflecting its ability to robustly model the disease–climate relationship. RF model identified temperature (T_min) as the most influential predictor, indicating that cooler night-time conditions may promote moisture retention and prolong surface wetness, thereby creating favourable conditions for bacterial survival and dispersal (Vidyadharan et al., 2025). Bayesian-optimized XGBoost showed enhanced accuracy relative to the baseline model, which is consistent with the observation by Zhou et al. (2023), who applied RF, LightGBM, and XGBoost models for predictive modelling, using SHAP analysis to interpret feature contributions, and reported that XGBoost exhibited the highest predictive performance among the models. Bayesian-optimized XGBoost achieved a 47% lower RMSE (0.823) compared to baseline XGBoost (1.560), representing substantial improvement in absolute prediction accuracy. Although R² declined from 0.698 to 0.536, this reflects the optimization objective (minimizing RMSE rather than maximizing R²) consistent with plant disease forecasting studies (Loona et al., 2025; Patil et al., 2025).

In the present study, SHAP analysis further enhanced model interpretability by quantifying the relative contribution of individual predictors, particularly highlighting key variables, such as WBT_Ngt, in influencing disease severity under field conditions. Collectively, these findings indicate that bacterial blight severity is governed by the combined effects of temperature and moisture dynamics across diurnal cycles rather than isolated meteorological variables. This approach enables robust early warning capabilities and supports data-driven management strategies, illustrating the added value of ML in predictive disease modelling beyond diagnostic applications. For validation time-series analysis an 80:20 temporal split (2013–2021 training, 2022–2024 test) was used. While expanding window cross-validation reduces look-ahead bias compared to random k-fold validation, it may still underestimate prediction error if disease dynamics shift abruptly due to emerging pathogen strains or changes in management practices.

Time-series analysis further supported the temporal patterns of bacterial blight severity observed in this study. The ARIMA model showed moderate forecasting performance, indicating that weekly disease severity followed a consistent temporal structure over the study period. Similar observations have been reported in long-term disease forecasting studies using ARIMA models on multi-year climatic and outbreak records, where reliable predictive accuracy demonstrated the suitability of ARIMA-based approaches for climate-responsive pest and disease management (Wang and Li, 2025). Although SARIMA modelling was applied to evaluate potential seasonal effects, the final model did not retain seasonal components, suggesting that disease progression was primarily governed by non-seasonal temporal dependence rather than distinct seasonal patterns. Similarly, Malik et al. (2025) applied ARIMA, SARIMA, and multivariate time-series models to decade-long climatic datasets and demonstrated their effectiveness in capturing seasonal variability and climate-driven disease dynamics, thereby supporting the relevance of integrated temporal modelling frameworks. Further improvement in biological interpretation was achieved using the VAR model, which jointly considered disease severity and meteorological variables. FEVD indicated that bacterial blight severity was predominantly governed by its intrinsic temporal dependence, reflecting strong disease persistence driven by recurrent infection cycles and continuous the production and dissemination of infectious propagules within the host population (Beresford et al., 2017). The IRF further demonstrated the delayed effect of temperature, indicating a lagged response consistent with biological processes such as pathogen multiplication, incubation period, and symptom expression. Such lagged responses of climatic variables have been reported in epidemiological studies, where temperature influences disease occurrence after a temporal delay rather than acting instantaneously (Torriani et al., 2025). Together, these findings demonstrate that bacterial blight development is governed by a combination of temporal persistence and environment-mediated modulation, where internal disease dynamics drive progression, and meteorological conditions regulate the rate and intensity of infection processes.

While the present study successfully integrates statistical, time-series, and ML approaches to assess the temporal and seasonal dynamics of bacterial blight using 10 years of data, future research could expand to spatiotemporal analyses to capture geographic variations in the disease incidence. Incorporating advanced AI and deep learning architectures, along with ensemble modelling, can further enhance predictive accuracy and early warning capabilities. The integration of GIS-based spatial mapping, directional trend analysis, and partial temporal modelling can support more precise forecasting, enabling improved decision-making and adaptive pest management strategies for pomegranate cultivation.

## 5 Conclusion

This study integrated statistical analysis, time-series modelling, and ML regression models to evaluate and forecast bacterial blight severity in pomegranates across nine districts in Maharashtra from 2013 to 2024. Correlation analysis identified weak positive associations with temperature (T_max) and moderate negative associations with RH (aft), whereas ARIMA, SARIMA, and VAR models captured temporal dependency and weekly disease progression patterns. Among the ML approaches, LightGBM exhibited the highest predictive accuracy for complex nonlinear disease and weather interactions. These insights underscore the value of climate-responsive predictive modelling in advancing bacterial blight risk management for pomegranate cultivation. These outcomes can be translated into practice through a web-based early warning system, enabling timely adaptive interventions and supporting sustainable disease management.

## Data Availability

The raw data supporting the conclusions of this article will be made available by the authors, without undue reservation.
