# Supplementary material for: Machine learning and time-series approaches for forecasting bacterial blight of pomegranate
Source: Front Plant Sci. 2026 Jul 16;17:1787474. doi: 10.3389/fpls.2026.1787474 (PMC13422461; doi:10.3389/fpls.2026.1787474)
Supplement: Supplementary file 1 [file Table1.docx]

**Supplementary Figures**


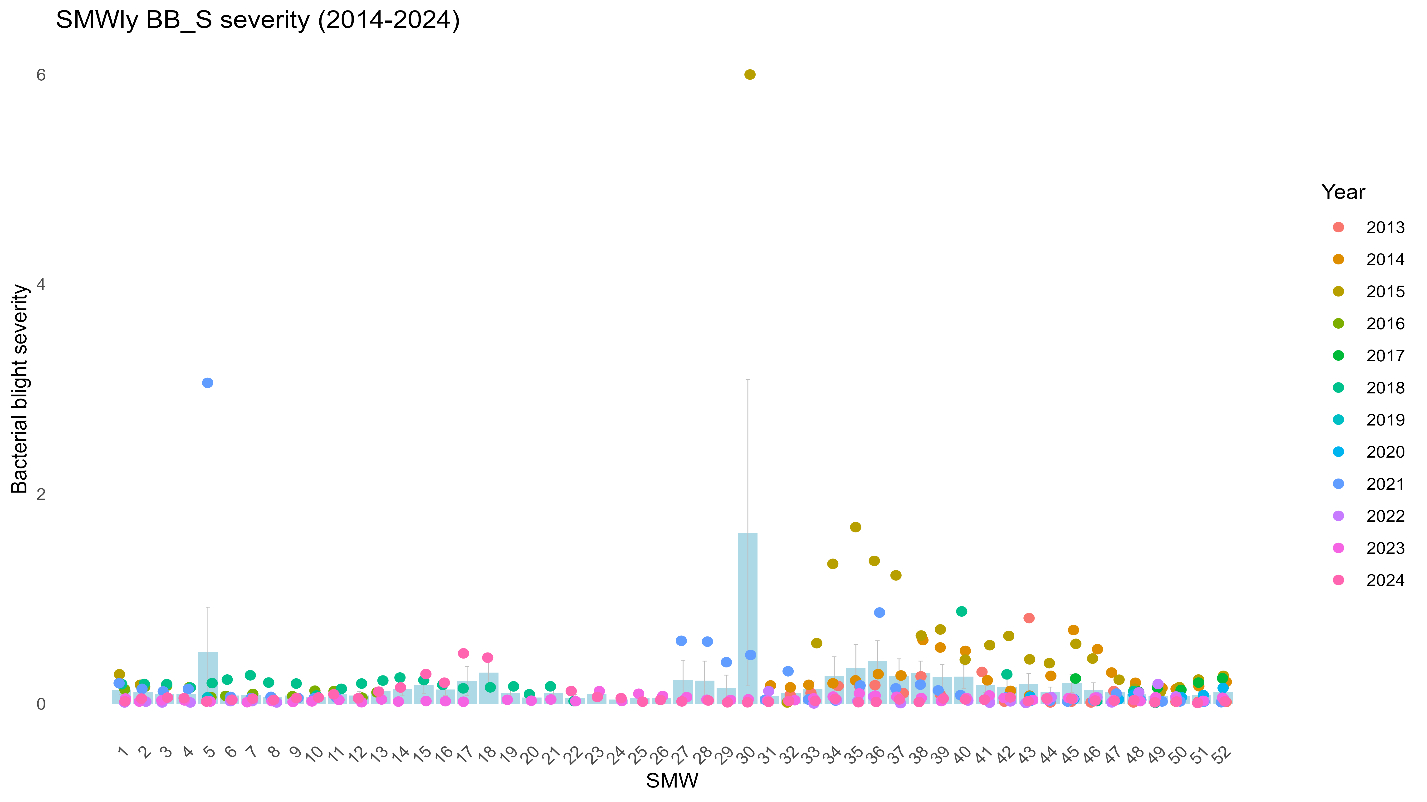


**Fig. S1** Combined bar and scatter plot showing SMW-wise mean bacterial blight severity (%) with inter-annual variability (2014–2024).


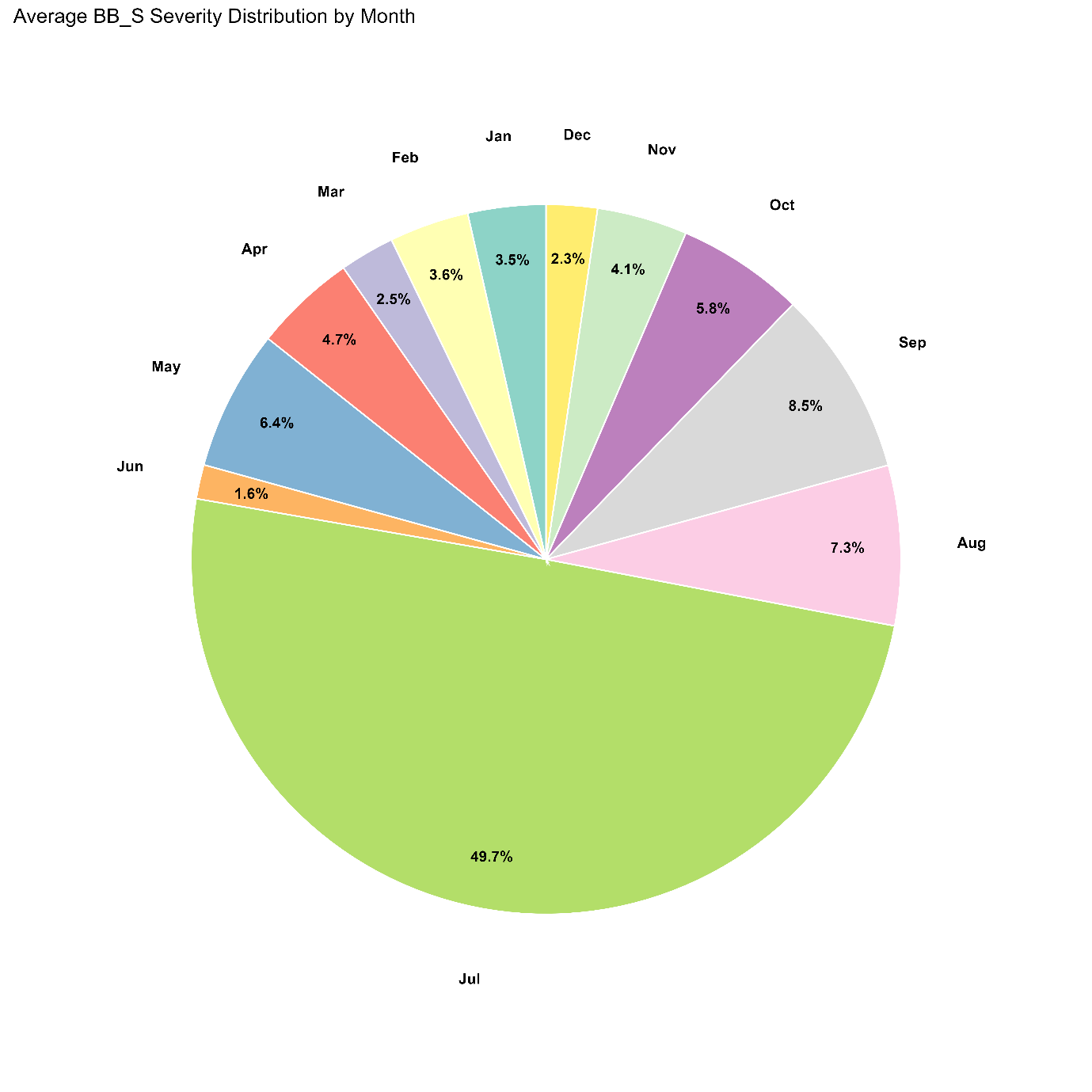
**Fig. S2** Pie chart representing the proportional contribution of each month to the average bacterial blight severity (%).


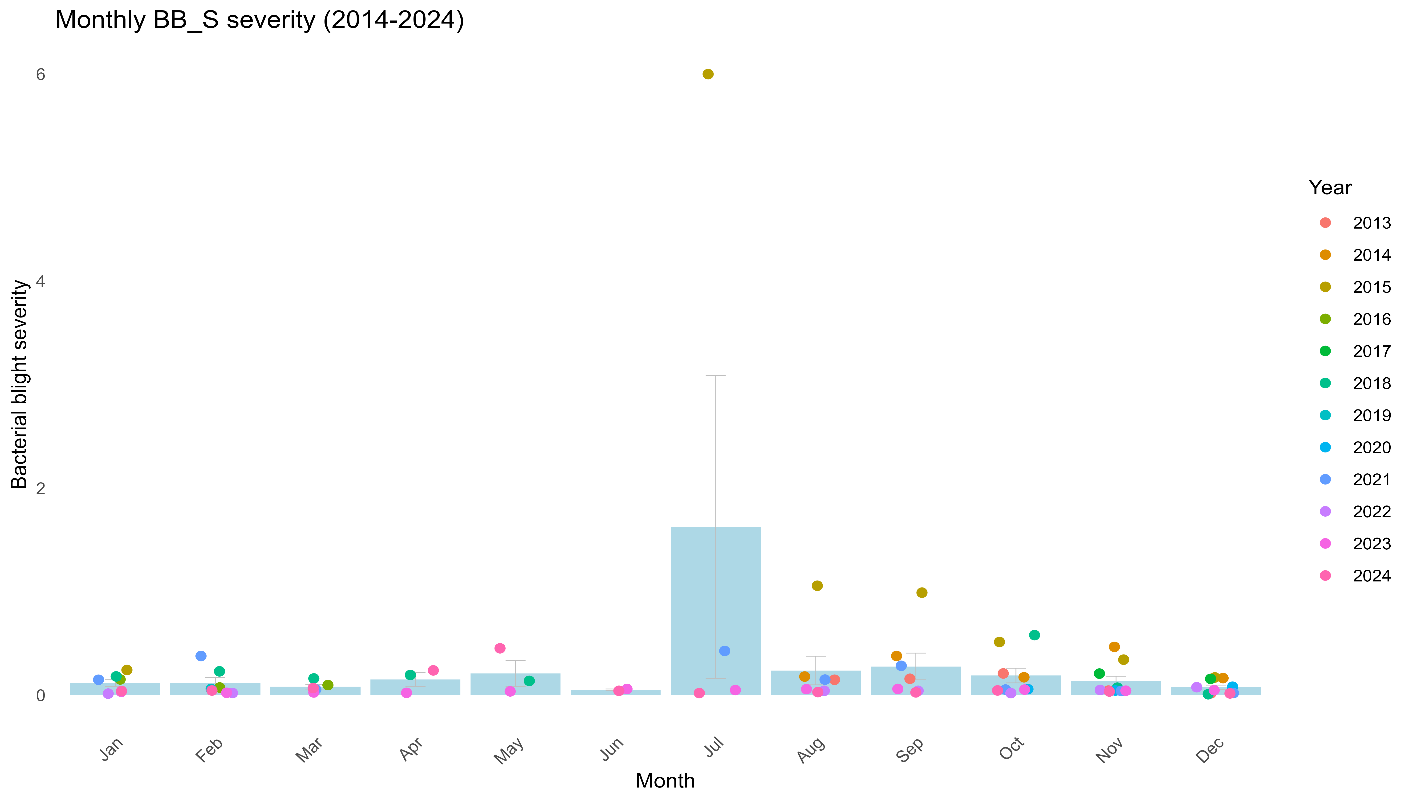


**Fig. S3** Bar plot displaying monthly mean bacterial blight severity (%) aggregated over the study period (2014–2024).


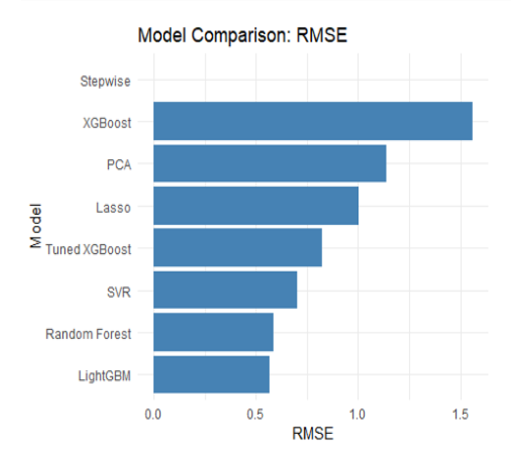


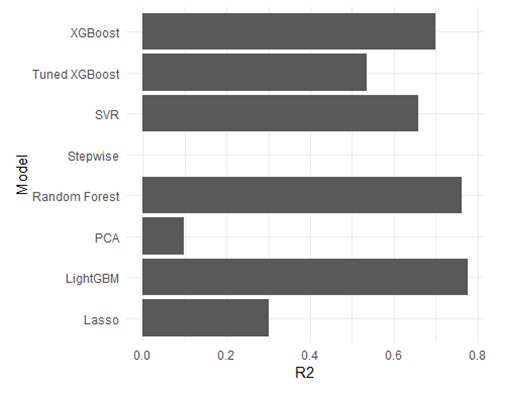

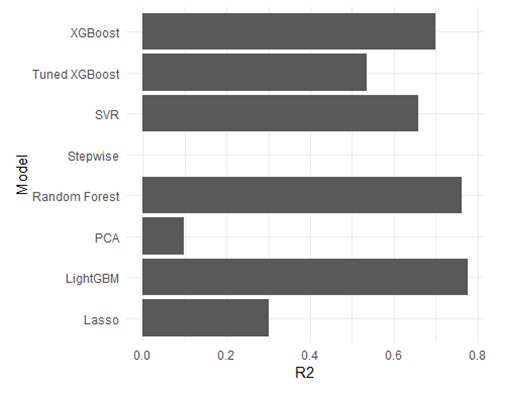


**Fig. S4** Comparative performance of ML based regression models evaluated using the RMSE and R^2^ values.


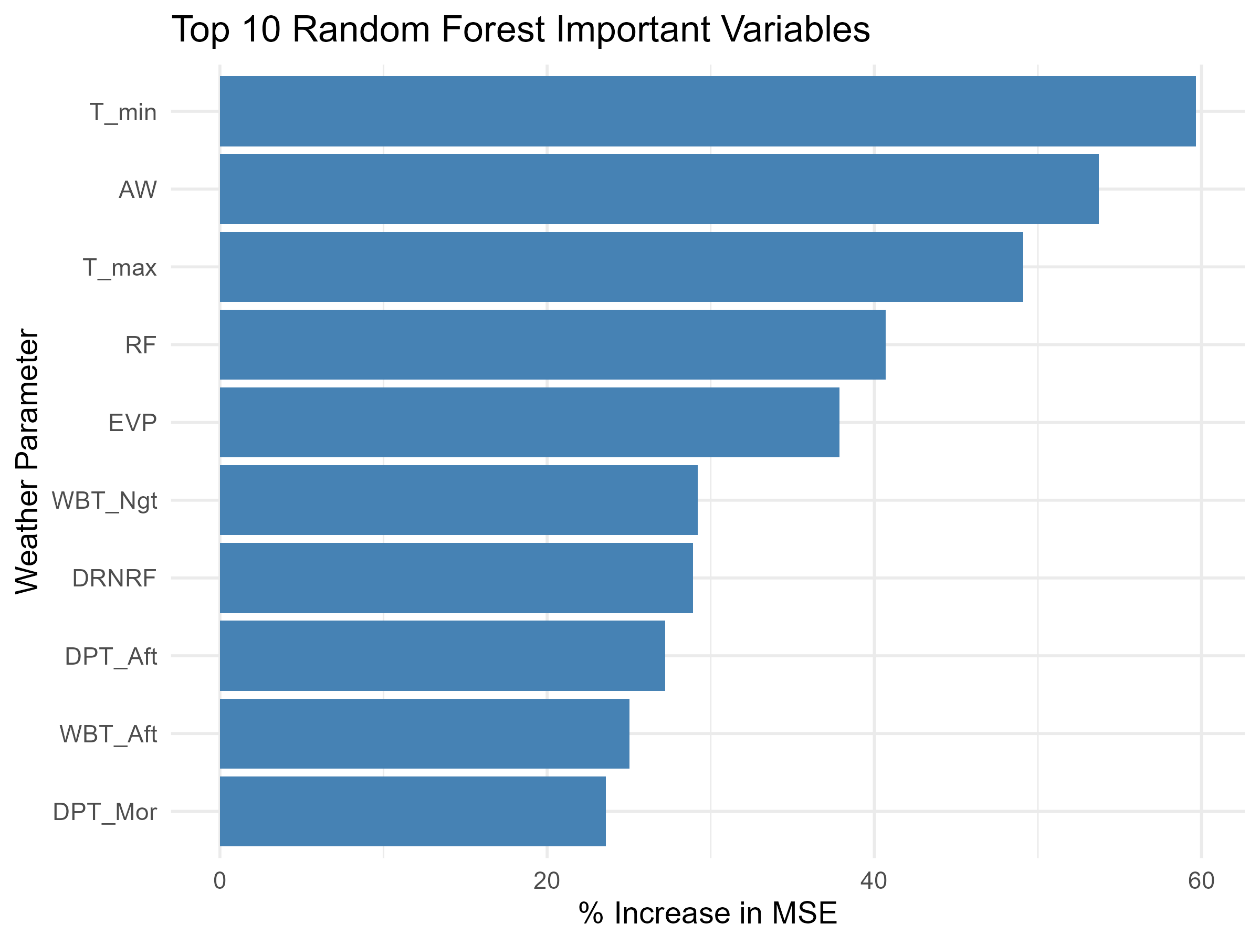


**Fig. S5** Random forest variables importance based on percentage increase in MSE.





**Fig. S6** Forecast Error Variance Decomposition (FEVD) plot derived from the VAR model showing the relative contribution of bacterial blight severity and associated meteorological variables.
